# Local detection of microvessels in *IDH*-wildtype glioblastoma using relative cerebral blood volume: an imaging marker useful for astrocytoma grade 4 classification

**DOI:** 10.1186/s12885-021-09117-4

**Published:** 2022-01-06

**Authors:** María del Mar Álvarez-Torres, Elies Fuster-García, Javier Juan-Albarracín, Gaspar Reynés, Fernando Aparici-Robles, Jaime Ferrer-Lozano, Juan Miguel García-Gómez

**Affiliations:** 1 Universitat Politècnica de València, Biomedical Data Science Laboratory, ITACA, 46022 Valencia, Spain; 2 Oslo University Hospital, Department of Diagnostic Physics, 0424 Oslo, Norway; 3 Health Research Institute Hospital La Fe, Department of Medical Oncology, Cancer Research Group, 46026 Valencia, Spain; 4 Health Research Institute Hospital La Fe, Department of Medical Imaging, 46026 Valencia, Spain; 5 Health Research Institute Hospital La Fe, Department of Pathology, 46026 Valencia, Spain

**Keywords:** Glioblastoma, Relative blood volume, DSC perfusion, Microvascular proliferation, IDH mutation, Histopathology

## Abstract

**Background:**

The microvessels area (MVA), derived from microvascular proliferation, is a biomarker useful for high-grade glioma classification. Nevertheless, its measurement is costly, labor-intense, and invasive. Finding radiologic correlations with MVA could provide a complementary non-invasive approach without an extra cost and labor intensity and from the first stage. This study aims to correlate imaging markers, such as relative cerebral blood volume (rCBV), and local MVA in IDH-wildtype glioblastoma, and to propose this imaging marker as useful for astrocytoma grade 4 classification.

**Methods:**

Data from 73 tissue blocks belonging to 17 *IDH-*wildtype glioblastomas and 7 blocks from 2 *IDH*-mutant astrocytomas were compiled from the Ivy GAP database. MRI processing and rCBV quantification were carried out using ONCOhabitats methodology. Histologic and MRI co-registration was done manually with experts’ supervision, achieving an accuracy of 88.8% of overlay. Spearman’s correlation was used to analyze the association between rCBV and microvessel area. Mann-Whitney test was used to study differences of rCBV between blocks with presence or absence of microvessels in IDH-wildtype glioblastoma, as well as to find differences with *IDH*-mutant astrocytoma samples.

**Results:**

Significant positive correlations were found between rCBV and microvessel area in the *IDH*-wildtype blocks (*p* < 0.001), as well as significant differences in rCBV were found between blocks with microvascular proliferation and blocks without it (*p* < 0.0001). In addition, significant differences in rCBV were found between *IDH*-wildtype glioblastoma and *IDH*-mutant astrocytoma samples, being 2–2.5 times higher rCBV values in *IDH*-wildtype glioblastoma samples.

**Conclusions:**

The proposed rCBV marker, calculated from diagnostic MRIs, can detect in *IDH*-wildtype glioblastoma those regions with microvessels from those without it, and it is significantly correlated with local microvessels area. In addition, the proposed rCBV marker can differentiate the IDH mutation status, providing a complementary non-invasive method for high-grade glioma classification.

**Supplementary Information:**

The online version contains supplementary material available at 10.1186/s12885-021-09117-4.

## Background

*IDH*-wildtype glioblastoma is the most lethal and common tumor of the central nervous system, resulting in a median prognosis of 12–14 months [1, 2] and being characterized by its high and heterogeneus vascularity [3–5]. Blood supply is required for the establishment, growth, and progression of the tumor; and several mechanisms are implicated in the formation of new vessels [3–5]. One of the results of these mechanisms is microvascular proliferation (MVP), which generally occurs in the core of glioblastomas by sprouting new vascular microvessels from pre-existing ones, depending on the presence of hypoxia [3].

These pathologic heterogeneity features, including vascular proliferation, robust angiogenesis and extensive microvasculature heterogeneity could vary depending on *IDH*-mutation status in high-grade gliomas [6]. In fact, the last update of 2020 CNS glioma classification and grading [2] differentiates between *IDH*-wildtype glioblastomas and *IDH*-mutant astrocytomas (previously named as IDH-mutated glioblastoma) as different type of gliomas, with different prognosis and vascular characteristics.

MVP is together to necrosis, the first criterion in the last update of 2020 CNS glioma classification and grading [2]. It is marked by two or more blood vessels sharing a common vessel wall [5], and interactions between tumor cells and blood vessels during microvascular proliferation seem to facilitate tumor growth [5–8]. The result of MVP is the formation of large-lumen microvessels, usually with a glomeruloid appearance, that represent one of the main histopathologic hallmark of glioblastoma [9].

Considering the relevance of this vascular process, the microvessel area (MVA), i.e., the total area covered by the microvessels in the tumor sample, and microvessel density (MVD), i.e., the number of microvessels per volume unit, have been previously investigated [9–16]. Different studies suggest that MVD poorly describes the morphometric diversity of these microvessels in high-grade gliomas [9–11]. However, MVA may provide a more robust clinical biomarker, useful for prognosis and grading [9–11, 17–21]. Regardless of this evidence, the histopathological quantification of MVA is still used exclusively in the research setting. Relevant limitations, including time- and cost-expending, labor intensity, and invasiveness make it challenging for routine clinical practice.

A complementary approach to overcom1e the limitations in MVA quantification is perfusion MRI [9, 12]. Some studies found that measures of relative cerebral blood volume (rCBV) positively correlate with microvascular structures in different glioma tumors [9, 11, 13–16]. However, these studies are few and present important limitations such as animal-based studies [11, 13], small cohorts of glioblastoma patients [9, 14–16], low number of analyzed histopathological specimens [9, 14–16], or analysis with non-spatial coregistered data [13, 20].

The integration of advanced and automatic techniques capable of calculating robust imaging markers, including rCBV, could help in high-grade glioma classification, including the diagnosis of *IDH*-wildtype glioblastoma and *IDH*-mutant astrocytoma. Besides, the complementary use of rCBV would involve important advantages since calculations derived from routine presurgical MRIs can be performed through automatic and robust methodologies, such as ONCOhabitats methodology [22–24].

In this context, we hypothesize that MVA could be directly associated with the rCBV in *IDH*-wildtype glioblastoma and this correlation can be measured using a robust MRI processing service. The areal density of microvessels on sections is an unbiased estimator of the volume density of microvessels according to the Delesse principle [25], and we hypothesize that the volume of microvessels can be related to the rCBV. Since the typical spatial resolution, DSC sequences is 2-mm in-plane × 5-mm slices [26], the calculation of rCBV would be reliable when it is calculated in areas larger than 2 mm.

In addition, we hypothesize that rCBV could be useful to find vascular differences between *IDH*-wildtype glioblastomas and *IDH*-mutant astrocytomas [6], and therefore, supporting the new glioma classification, which differentiates between these two tumors, providing an imaging method based on routinary presurgical MRI and Artificial Intelligence techniques.

The general purpose of our study is to evaluate the potential use of rCBV, calculated with the ONCOhabitats methodology, to detect the presence or absence of microvessels in different regions of *IDH-* wildtype glioblastoma, and to find differences in vascularity between *IDH*-wildtype glioblastoma and *IDH*-mutant astrocytoma. The study’s specific objectives are 1) to analyze the histopathologic and radiologic correlation between the imaging markers (rCBV_mean_ and rCBV_max_) with the local MVA in *IDH*-wildtype glioblastoma; 2) to study whether these imaging markers can differentiate regions of the tumor with presence or absence of microvessels 3) to analyze the capacity of the rCBV to differentiate between *IDH*-wildtype glioblastoma and *IDH*-mutant astrocytoma samples.

## Methods

### Clinical data collection

**Fig. 1:**
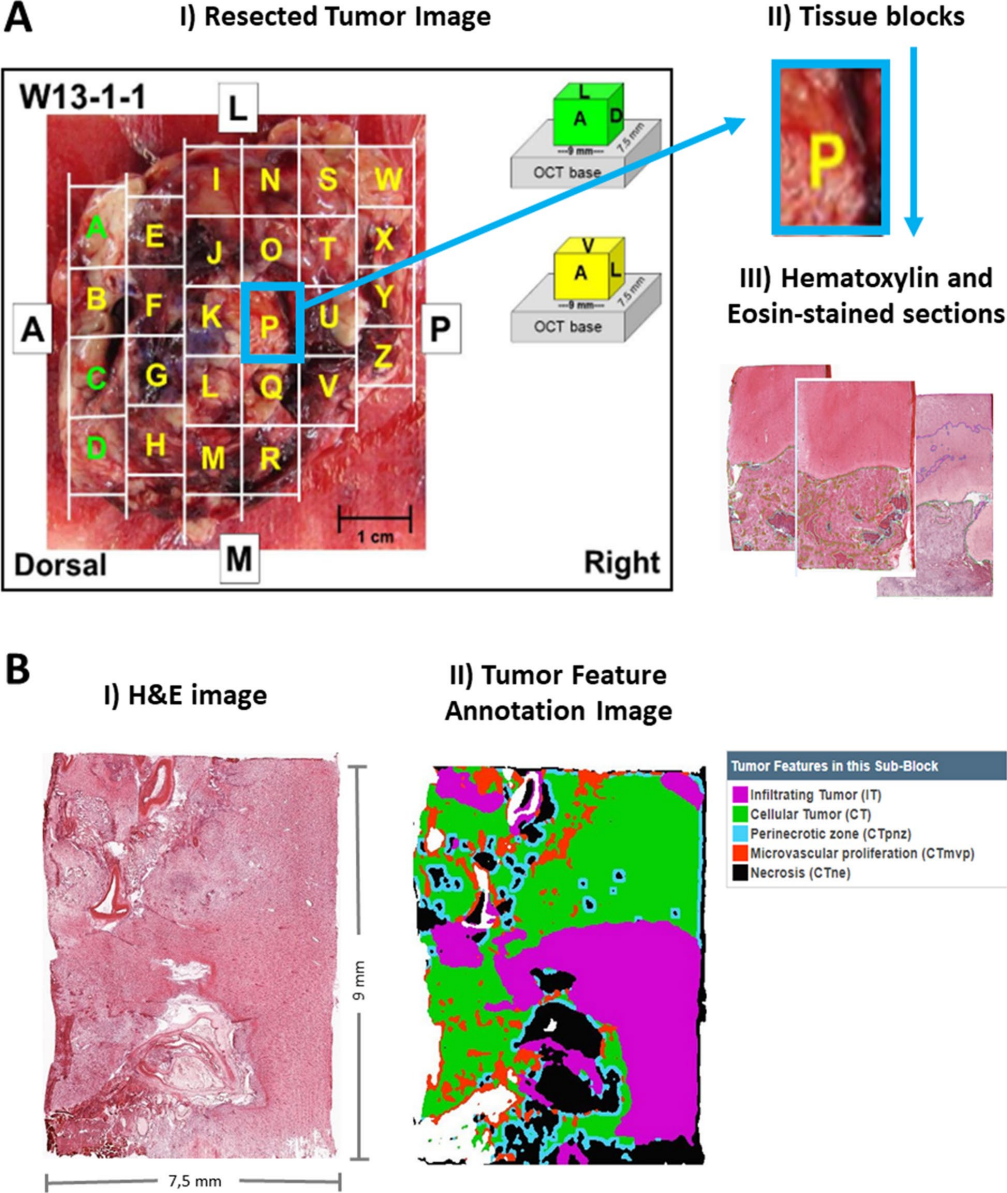
**A**: Included information in the Ivy Glioblastoma Atlas Project (Ivy GAP) database: I) Resected Tumor Image II) divided in tissue blocks; and III) Histopathological images at cellular resolution of hematoxylin and eosin-stained sections. **B**: I) Example of an H&E image of a slide from a resected block. II) The same image labeled with the different tissues and structures. Microvascular proliferation corresponds with areas in red color. (Images from Ivy GAP database [27], patient W55, block F, slice F.02). * OCT: Optimal Cutting Temperature compound. The term ‘OCT base’ is used to refer to the formed block after frozen and before sectioning

**Fig. 2:**
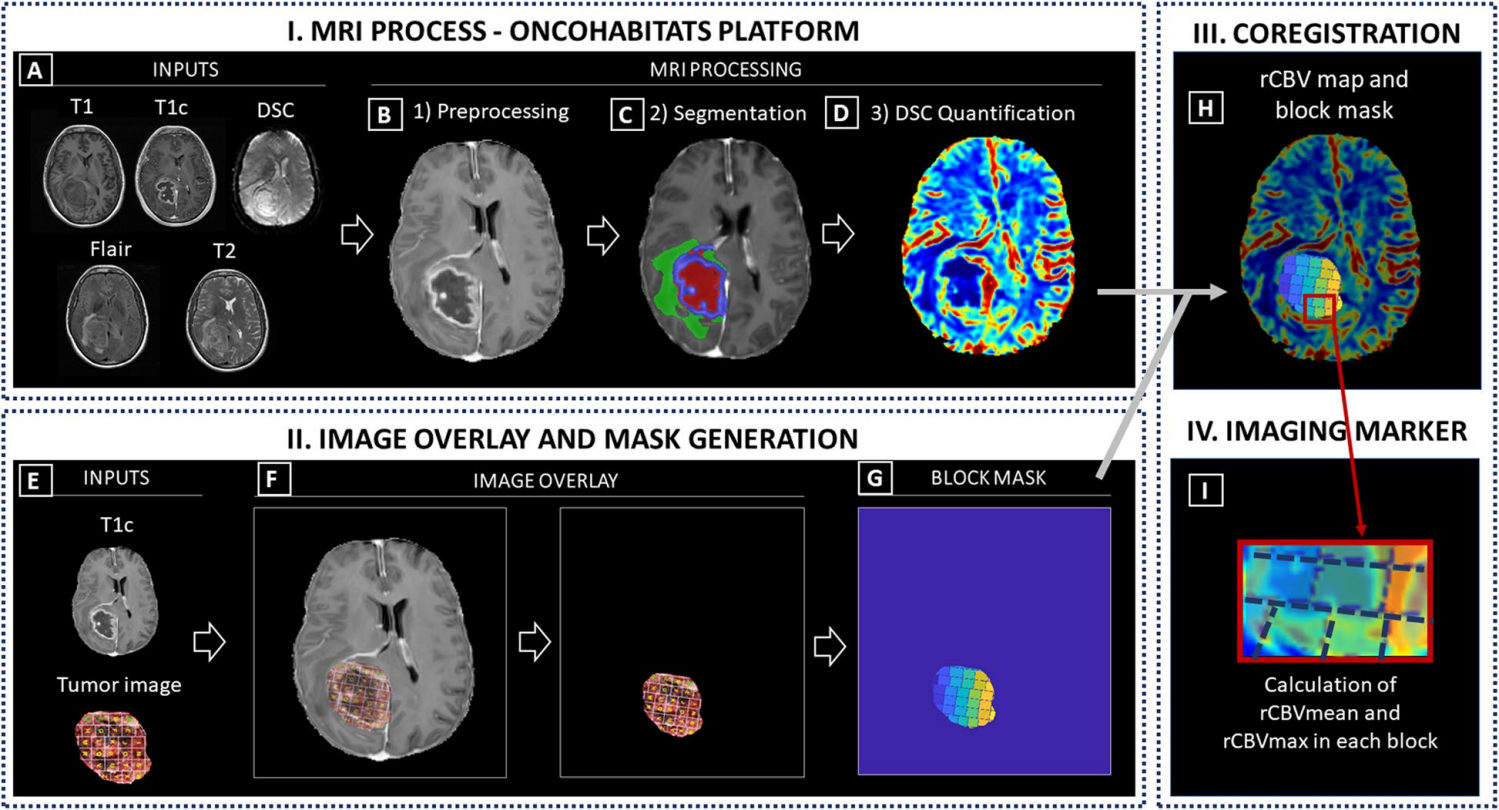
Phases of methodology: I) MRI Process conducted by the Segmentation Service included in the ONCOhabitats platform; II) Image overlay and mask generation; III) Coregistration and IV) Calculation of imaging markers. **A** Required inputs for the MRI process (T1, T1c, T2, Flair and DSC); (**B**) Preprocessing of the MRIs; (**C**) Classic segmentation of the lesion into tumor, edema and necrosis; (**D**) DSC perfusion quantification and rCBV map calculation. **E** Inputs to generate the block mask; (**F**) Image overlay process for mask generation; (**G**) Block mask; (**H**) Overlay of the rCBV map and block mask; (**I**) Calculation of imaging markers (rCBV_mean_ and rCBV_max_) in each tissue block

**Fig. 3:**
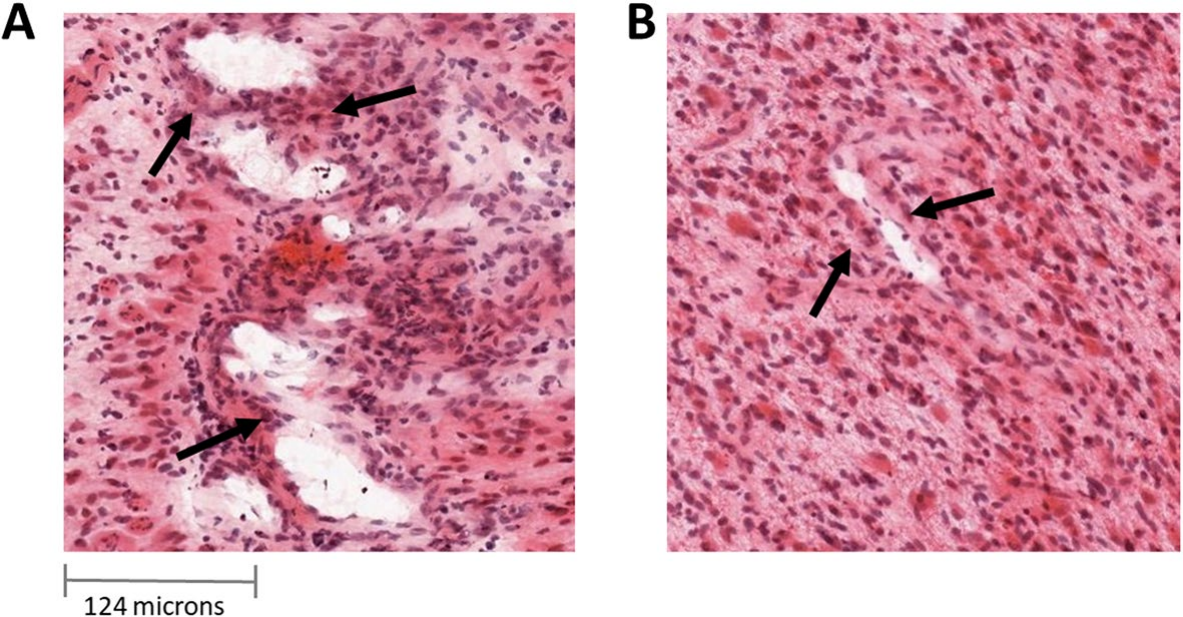
**A**: Aberrant vessels generated by microvascular proliferation sharing common vessel walls of endothelial and smooth muscle cells. **B**: Normal vessels. (Images from Ivy GAP database)

The Ivy Glioblastoma Atlas Project (Ivy GAP) database (www.glioblastoma.alleninstitute.org) [26] was used for this study since it includes: (i) Presurgical MRI data, including DSC perfusion sequences, (ii) histopathological data labeled, including microvessel area, (iii) images of the complete resected tumors with the blocks marked, and (iv) information about *IDH* mutation status. This public database includes 41 verified patients with astrocytoma grade 4, with a total of 42 tumors, with the following information per tumor: 1) images of the resected tumor (Fig. 1A.I) divided into tissue blocks (Fig. 1A.II); 2) histopathological images at a cellular resolution of hematoxylin and eosin-stained sections (collected from the tissue blocks) annotated for anatomic structures, including areas of microvessels (Fig. 1A.III and Fig. 1B), and 3) pre-surgical MRI studies of the patients: including pre and post-gadolinium T1-weighted MRI, T2-weighted MRI, FLAIR and Dynamic Susceptibility Contrast (DSC) T2* perfusion-weighted MRI (Fig. 2A).

### Patient and tumor selection

#### Inclusion criteria

Inclusion criteria for patients participating in the study were: *i)* histopathological confirmation of astrocytoma grade 4 (*IDH*-wildtype glioblastoma or *IDH*-mutant astrocytoma)*; ii)* access to complete MRI studies, including pre and post-gadolinium T1-weighted (T1 and T1c, respectively), T2-weighted, FLAIR T2-weighted, and DSC T2* perfusion sequences (Fig. 2A); *iii)* access to the resected tumor image (Fig. 1A); and *iv)* approval by an expert radiologist and histopathologist of the correct overlay/registration of the image of the resected tumor and the MRI of each patient (Fig. 2).

According to 2020 update classification of the central nervous system [1], we consider *IDH*-wildtype glioblastoma and *IDH*-mutant astrocytoma as different tumors.

#### Exclusion criteria

Exclusion criteria for patients were: *i)* inability to correctly overlay the image of the resected tumor over the MRI; *ii)* tumor tissue segmentation error during the processing; *iii)* to present extensive hemorrhage that could affect to a correct quantification of perfusion maps.

#### *IDH*-mutant astrocytomas

From 3 *IDH*-mutant astrocytomas, 1 was not included because of defective T1c image, not allowing a correct process with the ONCOhabitats platform. Finally, 2 *IDH*-mutant glioblastomas were included in the study.

### MRI acquisition and processing

MR images were obtained on a 3.0 or 1.5 T scanner (18 and 1 patients, respectively)*.* More information about MRI data is included in Table S1 of the Supporting Information, and it is publicly available in The Cancer Image Archive (TCIA) [27]. MRI processing was carried out using the ONCOhabitats platform [24], freely available in https://www.oncohabitats.upv.es. The ONCOhabitats analysis included the following automatic stages (Fig. 2):

1. *MRI Preprocessing* (Fig. 3B), including voxel isotropic resampling of all MR images, correction of the magnetic field in homogeneities and noise, rigid intra-patient MRI registration, and skull-stripping (Fig. 2A).
2. *Astrocytoma grade 4 tissue segmentation* (Fig. 3C), performed by using an unsupervised segmentation method, which implements a state-of-the-art deep-learning 3D convolutional neural network (CNN), which takes as input the T1c, T2, and Flair MRI. The current CNN deployed in ONCOhabitats is the next iteration of our previously published one [24]. Therefore, right now it is an in-house development that incorporates to our previous networks the current state-of-the-art techniques in image segmentation, such as spatial- and channel-attention blocks, deep-multi-level losses or both custom training schedulers and residual- feature extraction blocks. The backbone of our network maintains the classical U-net architecture with long-term skip-connections but adding the previously mentioned mechanisms that significantly improve the segmentation’s quality. Moreover, a balanced training strategy that yields local patches of healthy, necrotic, edematous and enhancing tumor is adopted to ensure the network is not biased towards the highest prevalent tissue in the brain.

This method is based on Directional Class Adaptive Spatially Varying Finite Mixture Model, or DCA-SVFMM, which consists of a clustering algorithm that combines Gaussian mixture modeling with continuous Markov random fields to take advantage of the self-similarity and local redundancy of the images.

All the used networks are trained and tested with the public BRATS dataset (available at https://www.med.upenn.edu/sbia/brats2018/data.html), which currently consist of a corpus with more than 380 glioblastomas manually segmented by several expert radiologists. An evaluation oracle is also available to send the segmentation results over a test set to assess the quality of the network. Currently our results in term of Dice score are: 0.90 in the whole glioblastoma segmentation (necrosis + edema + enhancing tumor), 0.83 in the tumor core (necrosis + enhancing tumor) and 0.86 in enhancing tumor segmentation [24].

3. *DSC perfusion quantification* (Fig. 3D), which calculates the relative cerebral blood volume (rCBV) maps, as well as relative cerebral blood flow (rCBF) or Mean Transit Time (MTT), for each patient. In this phase, T1-weighted leakage effects are automatically corrected using the Boxerman method [28], while gamma-variate curve fitting is employed to correct for T2 extravasation phase. rCBV maps are calculated by numerical integration of the area under the gamma-variate curve. The Arterial Input Function (AIF) is automatically quantified with a divide and conquer algorithm.

A more detailed description of the methodology is included in Reference 24, and the results of a multicenter study demonstrated its robustness in included in Reference 22.

### Tissue processing and histological staining

All the information included in this section was collected from the Ivy GAP white paper, available in the Ivy GAP database (https://glioblastoma.alleninstitute.org/).

#### Tissue acquisition, subdivision, and freezing

Immediately after *en bloc* resection, each tumor was set on a surgical towel, rinsed with saline, and subdivided into 9 × 7.5 × 18 mm high (tumors W1-W12) or 9 × 7.5 × 9 mm high (tumors W13-W55) tissue blocks. Using custom-fabricated L bars, each block was supported for freezing from the bottom of the L bar assembly (top of tissue block) on a dry ice isopropanol bath. As the block was rapidly frozen, an OCT base was formed with a disposable cryomold, added to the bottom of the tissue block, and frozen with a freeze aerosol (Fig. 1A). The 18 mm high blocks were divided into two 9 mm pieces on a refrigerated dissection table (− 15C). The bottom (block .1) contained the original OCT base, whereas the top (block .2) was embedded in OCT at the chopped interface. Blocks were stored at -80C before processing.

#### Cryosectioning for standard in situ hybridization (ISH) and hematoxylin and eosin (H&E)

Fresh frozen tissue blocks were removed from − 80 °C, equilibrated at − 15 °C in cryostats, mounted on chilled chucks, and sectioned at 20 μm with object temperature of − 10 °C or − 11 °C to reduce chatter through the necrotic areas and folds on the leading edge that contacted the blade first.

#### ISH image acquisition and processing

Whole slides were scanned directly to SVS file format at a resolution of 0.5 μm/pixel without downsampling on ScanScope® scanners (Aperio Technologies, Inc.; Vista, CA) equipped with a 20x objective and Spectrum software. The raw image files of ~ 5 GB per image were archived after converting to JPEG 2000 file format. The preprocessed images were flipped along the horizontal axis, white balanced, and compressed at a rate of 0.8 to ~ 400 MB per image. During post-processing, colorized expression values or heat masks showing ISH signal intensity were generated, and the closest H&E stained image of the same specimen was calculated for each ISH section.

During the review of images, the automated bounding box overlay was manually adjusted if necessary, so that each of 8 bounding boxes per slide was placed over the corresponding tissue section, and images of slides with focus or image tile stich misalignments were re-scanned. Images were failed if artifacts compromised data analysis (e.g., mechanical damage, mounting medium bubbles, hybridization bubbles, and NBT/BCIP precipitated aggregates) associated with the corresponding tissue section.

### Image overlay, mask generation, and image markers

To compare the information obtained from the rCBV maps and the MVA obtained from histopathological images, we overlaid the T1c MRI images to the images of tumor resected pieces, including the histopathology blocks’ location (Fig. 2E) provided in the Ivy GAP dataset. This 2D registration was performed manually using the following methodology (Fig. 2F):

- a) Localizing the lesion on the MRI images based on the hyperintensity of the T1 contrast and with the help of the segmentation masks, which includes the delineation of the active tumor, the edema and the necrotic tissue.
- b) Defining the orientation of the resected tumor and its blocks using the photograph provided at the Ivy GAP along with the parameters of orientation, position, and axial slice provided as reference.
- c) Refining the position by taking into account the necrosis masks and vascularity values in the blocks.
- d*)* Validation of the proposed overlay by expert radiologist (FAR), oncologist (GR) and histopathologist (JFL).

Additionally, we remove the background and generate a block mask with each block area delimited (Fig. 2G).

To evaluate the accuracy of the co-registration technique, we used the Intersection over Union method, i.e. we measured the area proportion overlap between the resected tumor image and the MRI image. We achieved a mean accuracy of 88.8%, and a TRE of 11.2%. In all cases the % was higher than 75.0%, being 76.1% the minimum and 98.1% the maximum.

Once the blocks were coregistered with the MRI space (2H), we could obtain the imaging markers rCBV_mean_ and rCBV_max_ for each independent block (Fig. 2I). We used the rCBV markers based on previous studies [22–24, 29, 30] in which it has been demonstrated that rCBV is the most robust DSC-perfusion marker, as well as it is the most used to find correlations with clinical outcomes.

The ONCOhabitats processing results for the patients with complete pre-surgical MRI (T1, T1Gd, T2, FLAIR and DSC perfusion) are publicly available in Zenodo (https://zenodo.org/record/4704106#.YJu8GagzY2w) [31]. For each patient, we include a PDF report containing an analysis summary; two folders with the resulting morphological and perfusion images in MNI and native spaces; and a third folder with the transformation matrices.

### Study variables

From each tissue block, several slides with an area of approximately 9 × 7,5 mm were collected with their corresponding hematoxylin and eosin (H&E) images of the IVY Gap database. The areas of different histopathological tissues were delimited in these images (Fig. 1B) and quantified data was available, including the mean value of MVA per block and the total area of each section of the block.

In order to normalize the MVA according to the area of each section of the tumor, the MVA value was divided by the area of the section. In addition, each block contains information from different sections; therefore, for the statistical analyses, we used the normalized mean value of MVA for each block, calculated with the following formula (where MVA_bs_ is the MVA present in the section s of one particular block b where n sections are available):

$${\mathrm{MVA}}_{\mathrm{b}}= mean\left(\frac{MVA_{b1}}{Area_{b1}},\dots, \frac{MVA_{bs}}{Area_{bs}},\dots, \frac{MVA_{bn}}{Area_{bn}}\right)\ \left.{\upmu \mathrm{m}}^2\right)$$

Besides, each block was classified into two groups: (1) blocks with presence of microvessels (MVA > 0) and (2) blocks with absence of microvessels (MVA = 0). An example of the microvessels generated by microvascular proliferation derived from the tumor progression, as opposed to normal vessels, is illustrated in Fig. 3.

#### Histopathological and radiologic correlation between MVA and rCBV in *IDH*-wildtype glioblastoma

Spearman’s correlation test was performed to study the association between the rCBV_mean_ and rCBV_max_ with the microvascular proliferation area (numeric continuous variable). Spearman coefficients and derived *p*-values were calculated, and descriptive measures of both rCBV_mean_ and rCBV_max_ among the whole block samples (mean, standard deviation, median, and range).

#### Differences of rCBV according to the presence or absence of microvessels in *IDH*-wildtype glioblastomas

Mann-Whitney tests were conducted to analyze the differences of rCBV_mean_ and rCBV_max_ between the group with presence of microvessels, and the group with absence of microvessels. The descriptive measures of rCBV (mean, standard deviation, and range) for each MVP and non-MVP groups were included.

#### Differences in rCBV between *IDH*-mutant astrocytoma and *IDH*-wildtype glioblastomas

Mann-Whitney tests were conducted to find the differences of rCBV_mean_ and rCBV_max_ between *IDH*-wildtype and *IDH*-mutant glioblastoma blocks. The descriptive measures of rCBV (mean, standard deviation, and range) for each *IDH* population were included.

Kaplan Meier curves were represented to analyze differences in survival between patients with *IDH*-wildtype glioblastoma and patients with *IDH*-mutant astrocytoma. In addition, Log rank test was carried out to study the significance of this difference (*p* < 0.005).

## Results

### Included patients

From the 41 patients included in the Ivy GAP database, 3 of them presented *IDH*-mutant glioblastomas (W10, W31, and W35); and the 38 remaining patients presented *IDH*-wildtype glioblastomas.

#### *IDH*-wildtype glioblastomas

From 38 *IDH*-wildtype glioblastoma, 14 were not included because of incomplete MRI studies (W04, W06, W16, W19, W20, W21, W26, W27, W28, W32, W39, W45, W53, and W54); From the 24 included patients, 3 patients were discarded due to inability to correctly overlay the image of the resected tumor over the MRI (W08, W09, and W11). The remaining 21 patients were processed with the ONCOhabitats platform. Of these 21 cases, 3 were excluded due to glioblastoma tissue segmentation errors (W01, W03, and W22); and 1 patient was excluded because of extensive hemorrhage that prevented a correct quantification of perfusion maps in DSC-MRI (W50). Finally, 17 *IDH*-wildtype glioblastomas were included in the study.

#### *IDH*-mutant astrocytomas

From 3 *IDH*-mutant astrocytomas, 1 was not included because of defective T1c image, not allowing a correct process with the ONCOhabitats platform. Finally, 2 *IDH*-mutant astrocytoomas were included in the study.

Demographic, clinical and MRI-related data of the total of 19 patients is included in Table S2 of the Supporting Information.

### Included blocks

#### *IDH*-wildtype glioblastomas

A total of 124 blocks with complete information from the 17 *IDH*-wildtype glioblastomas were initially considered. To develop the statistical analyses, only those blocks with more than 25 % (> 25%) of tumor, defined by imaging segmentation, were selected. This criterion was based on the existence of blocks that mostly contain necrotic tissue and therefore were not suitable for the study due to their lack of vascularization. Seventy-three (73) tissue blocks formed the final study sample of *IDH*-wildtype glioblastomas.

#### *IDH*-mutant astrocytomas

Thirteen blocks were initially considered from 2 *IDH*-mutant glioblastomas. Following the same criterion than for IDH-wildtype tumors, finally 7 blocks formed the final study sample of *IDH*-mutant glioblastomas.

1. ***Histopathological and radiologic correlation between MVA and rCBV in IDH-wildtype glioblastomas***

Table 1 includes the calculation of mean, standard deviation, median and range for rCBV_mean_ and rCBV_max_ for the 73 tissue blocks from 17 *IDH*-wildtype glioblastomas. The results of the Spearman correlation analyses between the MVA and each imaging marker (rCBV_mean_ and rCBV_max_) are also included in Table 1.

**Table 1:** Descriptive measures of rCBV_mean_ and rCBV_max_ (mean ± standard deviation, median and range) for the whole sample (71 blocks) and Spearman correlation results of these imaging markers with the microvascular proliferation area

IDH-wildtype glioblastomas	Mean ± standard deviation	Median	Range [min, max]	Spearman correlation with MVA (Coefficient; *p*value)
rCBV_mean_	4.91 ± 270	4.60	[1.07, 13.42]	C = 0.38; ***p*** = 0.0008*
rCBV_max_	9.52 ± 4.95	8.55	[2.35, 24.41]	C = 0.42; ***p*** < 0.0002*

*MVA* microvessel area

Both rCBV_mean_ and rCBV_max_ showed a significant positive correlation with MVA. That is, regions of the tumor with higher rCBV present significantly larger microvessel areas.

**Fig. 4:**
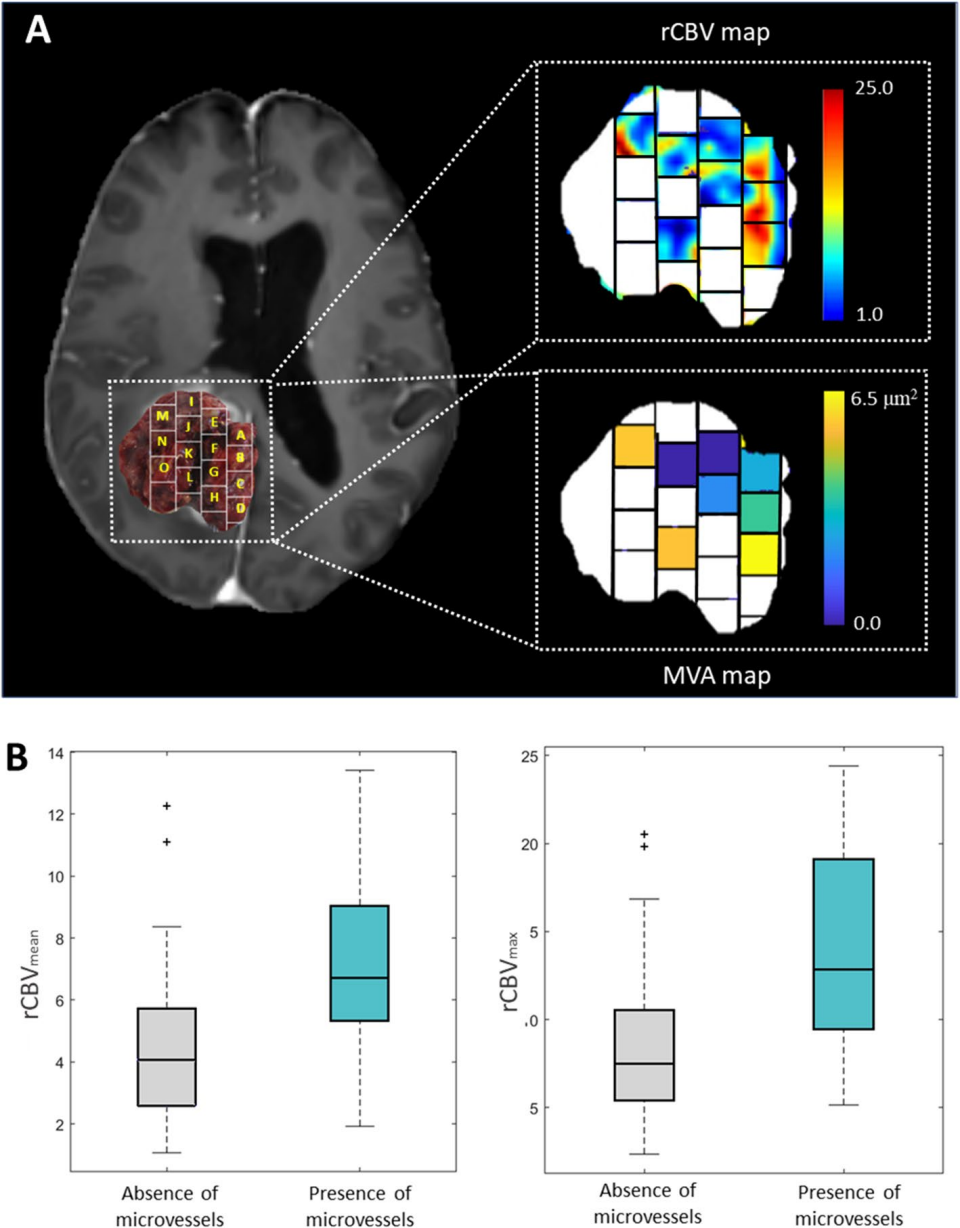
**A**: Post-gadolinium T1-weighted MRI overlaid with an image of the resected tumor including the delineation of the tissue blocks (left), example map of rCBV values (with a range of 1 to 25) of the areas occupied by the analyzed tumor blocks (top right), and example color map representing the MVA values (with a range from 0.0 to 6.5μ^2^) present in each analyzed block (below right). Blank blocks are not included due to lack of histopathological information. Example of patient W33 from the Ivy GAP database. **B**: Boxplot showing the significant differences of mean rCBV (left) and maximum rCBV (right) between the group with presence of microvessels and the group with absence of microvessels

Figure 4A shows an example (patient W33) of both rCBV and MVA maps to illustrate these two variables’ correlation. The white blocks did not present histopathological information, including the MVA data. It can be seen that blocks with areas with higher rCBV correspond with those with larger areas of MVA and vice versa.

(2) ***Differences in rCBV according to the presence or absence of microvessels in IDH-wildtype glioblastomas***

Table 2 shows the descriptive measures of rCBV_mean_ and rCBV_max_ for the blocks corresponding to the groups with presence and absence of microvessels*,* and the Mann-Whitney test results. All the measures (mean, minimum and maximum) of rCBV_mean_ and rCBV_max_ were higher in the group of blocks with presence of microvessels, presenting values two times superior to the group with no evidence of microvessels.

**Table 2:** Descriptive measures of rCBV_mean_ and rCBV_max_ (mean ± standard deviation and range) for the sample divided in groups according to the presence or the absence of microvessels, and Mann Whitney tests results from analyzing the differences of these imaging markers between these two groups are included

IDH-wildtype glioblastomas	*Blocks with Presence of Microvessels* (*n* = 14 blocks from 5 patients)		*Blocks with Absence of Microvessels* (*n* = 59 blocks from 12 patients)		MannWhitney test
	Mean ± std	Range [min, max]	Mean ± std	Range [min, max]	*p*-value
rCBV_mean_	7.08 ± 2.99	[1.92, 13.42]	4.40 ± 2.31	[1.07, 12.25]	***p*** = 0.0016*
rCBV_max_	14.15 ± 6.04	[5.15, 24,41]	8.43 ± 3.98	[2.35 20.50]	***p*** = 0.0005*

The Mann-Whitney test yielded significant differences (*p* < 0.05) between the rCBV_mean_ and the rCBV_max_ of the groups generated by the presence or absence of microvessels in *IDH*-wildtype glioblastomas. The rCBV_mean_ and rCBV_max_ can differentiate those regions of the tumor with microvessels.

Figure 4B shows the boxplots which illustrate these differences of the rCBV_mean_ and rCBV_max_ between the group with presence of microvessels (lower values) and the groups with absence of microvessels (higher values)*.*

(3) ***Differences in rCBV between IDH-wildtype glioblastomas and IDH-mutant astrocytoma***

Table 3 includes the Mann-Whitney test results, as well as the descriptive measures of rCBV_mean_ and rCBV_max_ for the tissue blocks corresponding to *IDH*-wildtype and *IDH*-mutant astrocytoma*.* Mean, median and maximum of rCBV_mean_ and rCBV_max_ were higher in the *IDH-*wildtype tissue blocks, presenting values more than two times superior to *IDH*-mutant tissue blocks. In addition, the Mann-Whitney test yielded significant differences (p < 0.05) in rCBV_mean_ and rCBV_max_ between *IDH*-wildtype and *IDH*-mutant glioblastomas.

**Table 3:** Descriptive measures of rCBV_mean_ and rCBV_max_ (mean ± standard deviation and range) for *IDH*-wildtype and *IDH*-mutant glioblastomas and Mann-Whitney tests results from analyzing the differences of these imaging markers between these two groups are included

	*IDH-wildtype tissue blocks* (*n* = 73)			*IDH-mutant tissue blocks* (*n* = 7)			Mann-Whitney test
	Mean ± std	Median	Range [min, max]	Mean ± std	Median	Range [min, max]	*p*-value
rCBV_mean_	4.91 ± 2.66	4.60	[1.07, 13.42]	2.29 ± 0.83	2.12	[1.49, 3.82]	***p*** = 0.0032*
rCBV_max_	9.52 ± 6.04	8.55	[2.35, 24,41]	3.98 ± 1.56	3.41	[2.56, 7.30]	***p*** = 0.0004*

**Fig. 5:**
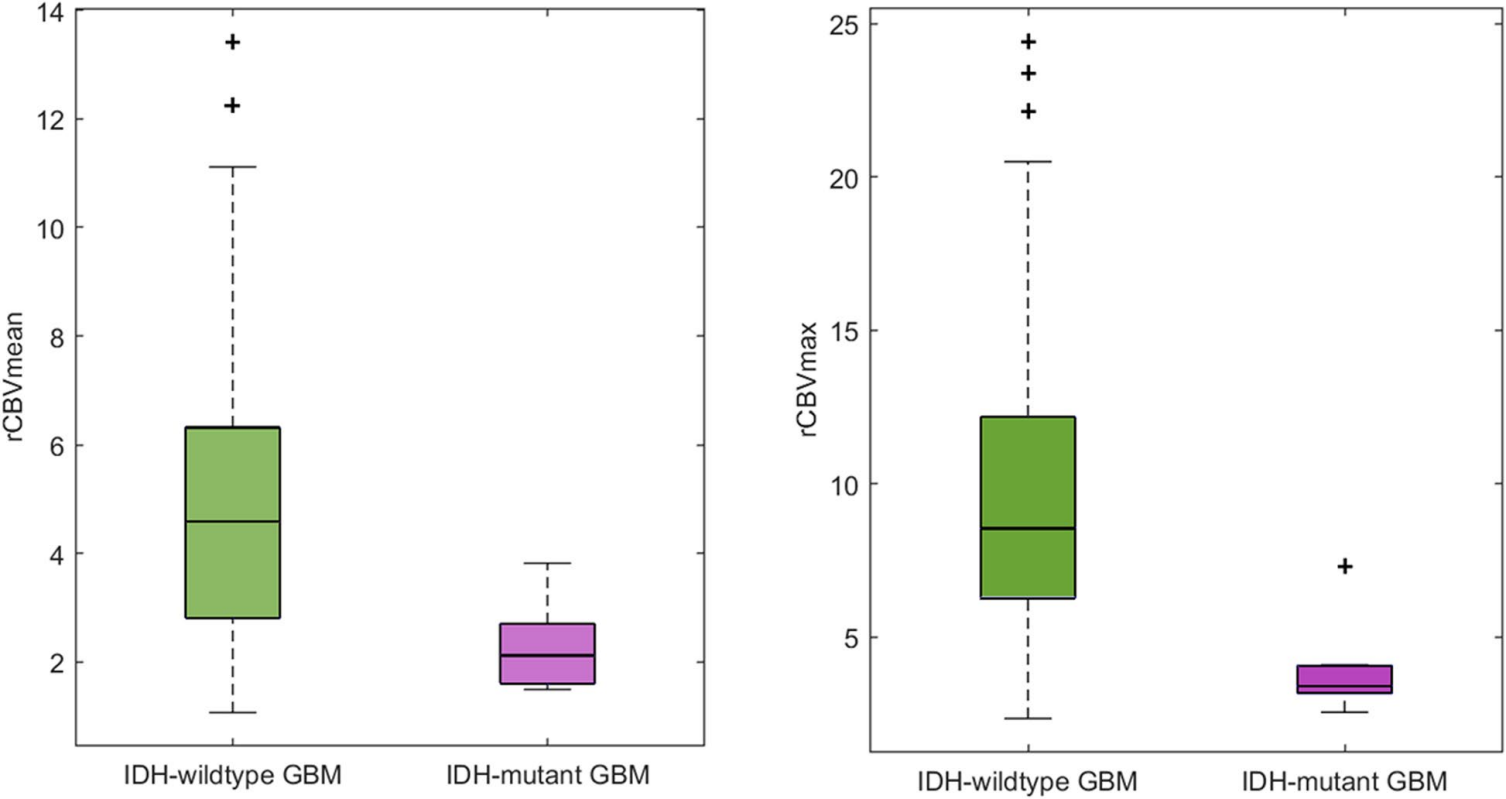
Boxplot showing the significant differences of mean rCBV (left) and maximum rCBV (right) between 73 *IDH-*wildtype tissue blocks from 17 patients and 7 *IDH*-mutant tissue blocks from 2 patients

Figure 5 shows the boxplots which illustrate the differences of the rCBV_mean_ and rCBV_max_ between the *IDH-*wildtype tissue blocks (higher values) and the *IDH-*mutant tissue blocks (lower values).

The Kaplan Meier curves with the estimated survival functions for patients with *IDH*-wildtype glioblastoma and *IDH*-mutant astrocyoma are included in Fig. S1 of the supporting information. Although differences in survival did not yield statistical significance (*p* = 0.099) between groups, estimated survival curves are completely separated, suggesting a substantial difference in survival times between patients with *IDH*-wildtype glioblastoma and *IDH*-mutant astrocyoma.

## Discussion

Microvascular proliferation is one of the main histopathologic hallmarks of glioblastomas, being key for the current glioma classification [1, 2]. In addition, microvessel area can be considered as an independent prognostic biomarker according to previous results, in which authors reported significant longer survival in patients with glioblastoma tumors lacking the presence of new microvessels [11, 17]. Those works suggested that tumoral microvasculature is associated with survival differences among tumors with identical histologic grades [9, 11, 17]. However, despite its clinical potential value, direct MVA quantification is clinically unfeasible due to its time-consuming and labor-intensive nature.

By contrast, imaging markers derived from routinary MRI protocols, such as rCBV, present several advantages since they are fast to calculate, it does not represent any extra cost, and it is non-invasive compared with MVA. However, although rCBV is used for the assessment of brain tumors, it is not widely considered as a biomarker for clinical decision-making yet, probably due to the difficulty to normalize the rCBV values, which can generate confusion about prospective clinical guidelines, but also due to the lack of robust studies using spatially localized histologic correlations [12].

In this sense, here we investigated the association between the imaging markers rCBV_mean_ and rCBV_max_, calculated with the validated method ONCOhabitats [22–24], and the MVA in *IDH*-wildtype glioblastoma samples. Moreover, we analyzed the differences of rCBV between those areas of the tumor with the presence of microvessels from those regions of the tumor without evidence of microvessels.

We found significant correlations between rCBV_mean_ and rCBV_max_ and MVA when analyzing 73 tissue samples derived from 17 human *IDH*-wildtype glioblastoma tumors. Also, we found significant results when we evaluated the differences of both rCBV_mean_ and rCBV_max_ between tissue blocks with presence of microvessels from those blocks defined by the absence of microvessels. Microvascular proliferation is, together to necrosis, the first criterion in the last update of 2020 CNS glioma classification and grading, since it is considered as one of the main hallmarks of high grade-gliomas (including *IDH*-wildtype glioblastoma and *IDH*-mutant astrocytoma). Therefore, its correlation with rCBV, makes it also a potential candidate useful in glioma classification. Despite the few similar studies conducted [9–16], our results are consistent with those previously reported, finding a significant positive correlation between rCBV and MVA and significant differences in rCBV in those regions of the tumor with presence or absence of microvessels. In previous studies developed with human data and which analyze continuous variables (MVA or MV) [14, 15], similar correlation coefficients were found (*ρ* = 0.42 [14]; *ρ* = 0.46 [15] vs. *ρ* = 0.43 in the present study). However, in previous studies, only 2 and 4 glioblastoma patients were enrrolled, versus the 73 samples derived from 17 patients used in the current study. Increasing the interpatient heterogeneity in our study resulted in not higher correlation coefficients. Nonetheless, the analyses are more robust and *p*-values more significant. A more detailed comparison with previous studies can be found in Table S3 of the Supporting Information.

Furthermore, in this study we have investigated the differences in rCBV_mean_ and rCBV_max_ between *IDH*-wildtype glioblastoma and *IDH*-mutant astrocytoma samples. We found that blocks from *IDH*-wildtype glioblastoma present almost 2.5 times higher rCBV values than blocks from *IDH*-mutant astrocytomas. These represent promising preliminary results to propose the rCBV, calculated with ONCOhabitats, to predict with a non-invasive method the *IDH* status in these gliomas and a complementary method for diagnosis.

This study has some limitations. Firstly, the manual registration between morphologic MRI images and the resected tumor image could be affected by deformations of the tumor tissue morphology when resected and/or the difficulty of finding matchings between both image features. Also, the number of independent analyzed samples is not much higher despite it being higher than in other previous studies. In addition, the results derived from the comparation between *IDH*-wildtype and *IDH*-mutant samples should be considered with caution, since only 7 blocks from 2 patients were included for the *IDH*-mutant group.

The results derived from this work suggest the potential of imaging vascular markers calculated with the ONCOhabitats platform for helping in unmet challenges in high-grade glioma management, including glioma classification and prediction of IDH mutation status, with a non-invasive method and from the initial stage of diagnosis. We consider that the rCBV is a clinically relevant option for decision making in glioblastoma [12, 29, 30], since it could be a complementary tool to histopathology for analyzing intratumor vascular heterogeneity at both temporal and spatial levels in a non-invasive way [23]. This marker could be especially relevant for inoperable tumors, for which an exhaustive histopathological analysis cannot be performed. An early diagnosis, a correct classification and a more precise and personalized analysis of the glioma will have a positive impact on the patient’s treatment. Furthermore, this study opens up the possibility of evaluating tumor vascularity more correctly after antiangiogenic treatments, in addition to other prognostic/predictive markers related to tumor vascularization.

In addition, we consider useful to provide the ONCOhabitats results for Ivy GAP dataset with the purpose of enabling researchers investigating other relevant correlations between imaging-based biomarkers and histopathology for prognostic/predictive applications in glioblastoma. These results are publicly available for viewing and downloading in Zenodo (https://zenodo.org/record/4704106#.YJu8GagzY2w) [31].

## Conclusions

The main conclusion of this study is the demonstration of a significant histopathological and radiologic correlation between the MVA and the rCBV in local regions of *IDH*-wildtype glioblastoma. The ONCOhabitats method allows a spatial location and detection of different regions of the tumor with presence of microvessels since the first diagnostic stage in a non-invasive way. In addition, significant differences in the rCBV values are found between *IDH-*wildtype glioblastomas and *IDH*-mutant astrocytomas, supporting the last update of glioma classification, which consider these gliomas as different tumors [1, 2].

## Supplementary Information

**Additional file 1: Table S1.** Information about the Magnetic Resonance Imaging (MRI) acquisition parameters. **Table S2.** Demographic, clinical and MRI-related data of included patients with IDH-wildtype glioblastoma (*n*=17) and IDH-mutant astrocytoma (*n*=2). **Table S3.** Comparative table with previous studies reported in literature related with the correlation between perfusion MRI and vascular features defined by histopathological analyses. **Figure S1.** Kaplan Meier curves with the estimated survival functions for IDH-wildtype and IDH-mutant glioblastoma patients from the Ivy GAP database included in the study.

## Data Availability

The histopathological and clinical data that support the findings of this study are openly available in the Ivy Glioblastoma Atlas Project at https://glioblastoma.alleninstitute.org/. The imaging data that support the findings of this study are openly available in Zenodo at 10.5281/zenodo.4704106. The methodology ONCOhabitats is publicly available for research use in www.oncohabitats.upv.es

## Abbreviations

IDH: Isocitrate dehydrogenase
MRI: Magentic Resonance Imaging
MVA: Microvessels area
rCBV: Relative cerebral blood volume
