# Supplementary material for: Local detection of microvessels in IDH-wildtype glioblastoma using relative cerebral blood volume: an imaging marker useful for astrocytoma grade 4 classification
Source: BMC Cancer. 2022 Jan 6;22:40. doi: 10.1186/s12885-021-09117-4 (PMC8734263; doi:10.1186/s12885-021-09117-4)
Supplement: Supplementary file 1 — Additional file 1: Table S1. Information about the Magnetic Resonance Imaging (MRI) acquisition parameters. Table S2. Demographic, clinical and MRI-related data of included patients with IDH-wildtype glioblastoma (n=17) and IDH-mutant astrocytoma (n=2). Table S3. Comparative table with previous studies reported in literature related with the correlation between perfusion MRI and vascular features defined by histopathological analyses. Figure S1. Kaplan Meier curves with the estimated survival functions for IDH-wildtype and IDH-mutant glioblastoma patients from the Ivy GAP database included in the study. [file 12885_2021_9117_MOESM1_ESM.docx]

**Supporting Information**

**Table S1:** Information about the Magnetic Resonance Imaging (MRI) acquisition parameters.

| MRI  Modalities | TR (ms) | TE (ms) | Flip angle | Slice Thickness (mm) | Number of temporal positions |
| --- | --- | --- | --- | --- | --- |
| T1c | 1940 | 10 | 12 | 5 |  |
| T2 | 3200 | 90 | 90 | 2-3 | - |
| FLAIR | 11000 | 140 | 90 | 2-3 | - |
| DSC | 1500 | 25 | 60 | 5 | 48 |

**Table S2**: Demographic, clinical and MRI-related data of included patients with *IDH*-wildtype glioblastoma (n=17) and *IDH*-mutant astrocytoma (n=2)

|  | *IDH*-wildtype glioblastoma | *IDH*-mutant astrocytoma |
| --- | --- | --- |
| Median age (years old) | 61 | 41 |
| Gender (#F;#M) | 8; 9 | 2; 0 |
| Initial KPS | 90 | 100 |
| Median Weight (kg) | 95.0 | 79.8 |
| Median Height (cm) | 173.0 | 173.0 |
| Tumor location (#right; #left) | 13; 4 | 1; 1 |
| #patients with complete chemotherapy/#patients with incomplete chemotherapy | 15/17 | 2/2 |
| #patients with complete radiotherapy/#patients with incomplete radiotherapy | 16/17 | 2/2 |

**Table S3**: Comparative table with previous studies reported in literature related with the correlation between perfusion MRI and vascular features defined by histopathological analyses.

|  | Human cohort | #Patients | #Tissue samples | Diagnostic MRI^a^ | Spatial corregistration^b^ | Continuous histopathologic variable^c^ |
| --- | --- | --- | --- | --- | --- | --- |
| Aronen HJ *et al.;* 1994 [14] | **✓** | 5 WHO IV (+14 other gliomas) | Not specified | ✘ | ✘ | ✘ |
| Cha S *et al*.; 2003 [17] | ✘ | 34 mice | 34 | NA | ✘ | ✘ |
| Chakhoyan *A et al.*; 2019 [18] | **✓** | 4 WHO IV (+7 WHO III) | Not specified.  1-3 per tumor | ✘ | **✓** | **✓** |
| Hu LS *et al.*; 2012 [8] | **✓** | 12 WHO IV (+12 other gliomas) | 21 | ✘ | **✓** | **✓** |
| Pathak AP *et al.;* 2001 [10] | ✘ | 12 rats with gliosarcoma | Over 3 slices per tumor | NA | **✓** | **✓** |
| Sadegui N *et al*.; 2008 [19] | **✓** | 2 WHO IV (+17 other gliomas) | 8 | ✘ | **✓** | **✓** |
| Sugahara T *et al*.; 1998 [20] | **✓** | 11 WHO IV (+19 other gliomas) | Not specified | ✘ | **✓** | ✘ |
| Present study | **✓** | 17 WHO IV | 73 | **✓** | **✓** | **✓** |

**Diagnostic MRI^a^**: Use of the initial MRI study performed as standard in the diagnosis of glioblastoma; **Spatial corregistration^b^**: Spatial corregistration between the MR images and the tissue samples; **Continuous histopathologic variable^c^:** Area/volume of microvascular structures; **NA**: Not aplicable.

**
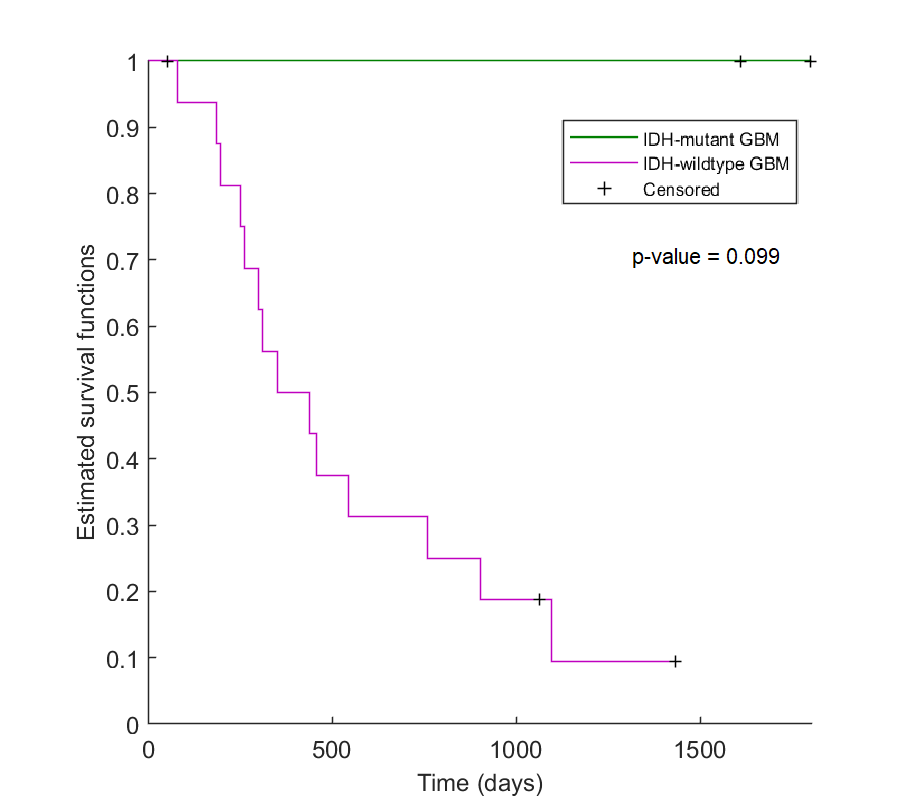
**

**Figure S1:** Kaplan Meier curves with the estimated survival functions for IDH-wildtype and IDH-mutant glioblastoma patients from the Ivy GAP database included in the study.
